# Pathogenic Variants in Selenoproteins and Selenocysteine Biosynthesis Machinery

**DOI:** 10.3390/ijms222111593

**Published:** 2021-10-27

**Authors:** Didac Santesmasses, Vadim N. Gladyshev

**Affiliations:** Division of Genetics, Department of Medicine, Brigham and Women’s Hospital, Harvard Medical School, Boston, MA 02115, USA; dsantesmassesruiz@bwh.harvard.edu

**Keywords:** selenium, selenoprotein, selenocysteine, genetic variance, human disease

## Abstract

Selenium is incorporated into selenoproteins as the 21st amino acid selenocysteine (Sec). There are 25 selenoproteins encoded in the human genome, and their synthesis requires a dedicated machinery. Most selenoproteins are oxidoreductases with important functions in human health. A number of disorders have been associated with deficiency of selenoproteins, caused by mutations in selenoprotein genes or Sec machinery genes. We discuss mutations that are known to cause disease in humans and report their allele frequencies in the general population. The occurrence of protein-truncating variants in the same genes is also presented. We provide an overview of pathogenic variants in selenoproteins genes from a population genomics perspective.

## 1. Introduction

Selenium is an essential trace element in mammals. Its main biological functions are mediated by selenoproteins, which contain selenium in the form of the 21st amino acid selenocysteine (Sec). Selenoproteins are important oxidoreductase enzymes widely conserved across mammals [1]. They are involved in diverse molecular pathways, with Sec typically found at the catalytic site [2]. Sec is incorporated into selenoproteins in response to a UGA codon, normally a stop codon, through a recoding mechanism that is selenium-dependent and involves a dedicated machinery [3]. There are 25 known selenoprotein genes in humans, and 24 in mice [4]. Mouse models have been particularly instrumental in interrogating the functions of selenoproteins and assessing their gene essentiality [5]. Five selenoproteins have been reported to be essential in mice: Gpx4 [6,7], Txnrd1 [8], Txnrd2 [9], Selenot [10], and Selenoi [11]. In humans, the significance of selenium and selenoproteins for health is manifested by inherited congenital disorders caused by mutations that disrupt selenoprotein synthesis or affect individual selenoproteins.

Here we provide an overview of clinically relevant genetic variants based on the analysis of the literature and specialized databases. We used ClinVar, a public archive of reports of the relationships of genetic variation and phenotypes with assessment of clinical relevance of variants submitted by researchers and genetic testing labs [12], and gnomAD, an aggregate of exome and genome sequencing data of unrelated individuals sequenced as part of various disease-specific and population genetic studies [13].

## 2. Pathogenic Variants in Selenoproteins

Selenoproteins associated with human syndromes thus far include 5 proteins: SELENON, GPX4, TXNRD1, TXNRD2, and SELENOI. Disruption of the selenoprotein synthesis pathway, which causes generalized selenoprotein deficiency, has been associated with mutations in SEPSECS, SECISBP2, and Sec tRNA (*TRU-TCA1-1*). The consequences of selenoprotein deficiency and causal mutations have been reviewed recently [14,15,16,17]. ClinVar currently lists a total of 72 pathogenic or likely pathogenic variants in selenoprotein genes, and 35 in the Sec synthesis machinery factors. 44 of those variants are observed in at least one individual in gnomAD.

### 2.1. SELENON

Selenoprotein N (SELENON, also known as SEPN1) is a transmembrane protein located in the endoplasmic reticulum (ER) [18]. Initially described by the in silico identification of its SECIS element [19], SELENON was shortly after linked to a congenital rigid spine muscular dystrophy [20], becoming the first selenoprotein associated with human disease. Its protein sequence contains an EF-hand domain and a Sec residue as part of a SCUG motif, where U corresponds to Sec, reminiscent of the Sec-containing motif in thioredoxin reductases. Since its association with disease, there has been a lot of interest in the characterization of its functions in the muscle [21,22]. A recent study showed how SELENON functions as a calcium sensor through its calcium-binding EF-hand domain and activates sarco/endoplasmic reticulum calcium ATPase (SERCA2)-mediated calcium uptake into the ER in a redox-dependent manner [23]. Knockout mice were also developed [24,25].

SELENON-related myopathy (SELENON-RM, formerly SEPN1-RM) is a congenital disorder caused by loss-of-function variants in the SELENON gene. SELENON-RM comprises four neuromuscular disorders initially described separately as rigid spine muscular dystrophy [20,26], multi-minicore disease [27], congenital fiber type disproportion [28], and desmin-related myopathy with Mallory body-like inclusions [29]. The clinical phenotype is characterized by early-onset axial muscle weakness, spinal rigidity, and scoliosis, with respiratory failure. Transmission is autosomal recessive, and patients are homozygous or compound heterozygous. To date, many genetic variants that disrupt SELENON, or prevent Sec insertion, have been identified in SELENO-RM patients [30]. ClinVar currently lists 65 variants in SELENON as pathogenic/likely pathogenic. Notably, those variants include a change in the Sec codon TGA to TAA [20], which produces a premature stop codon (currently erroneously classified as synonymous in ClinVar); a variant that affects the conserved quartet core of the SECIS [31]; and several variants in the Selenocysteine Redefinition Element (SRE), a small RNA hairpin loop adjacent to the UGA codon [20,32]. In those cases where Sec synthesis efficiency is reduced, transcript levels have been shown to be decreased, suggesting that the mRNA is targeted by nonsense-mediated decay [31,32]. The remaining pathogenic variants are missense changes in conserved residues, small insertions and deletions that lead to frameshift, and splice donor/acceptor variants.

**Figure 1:**
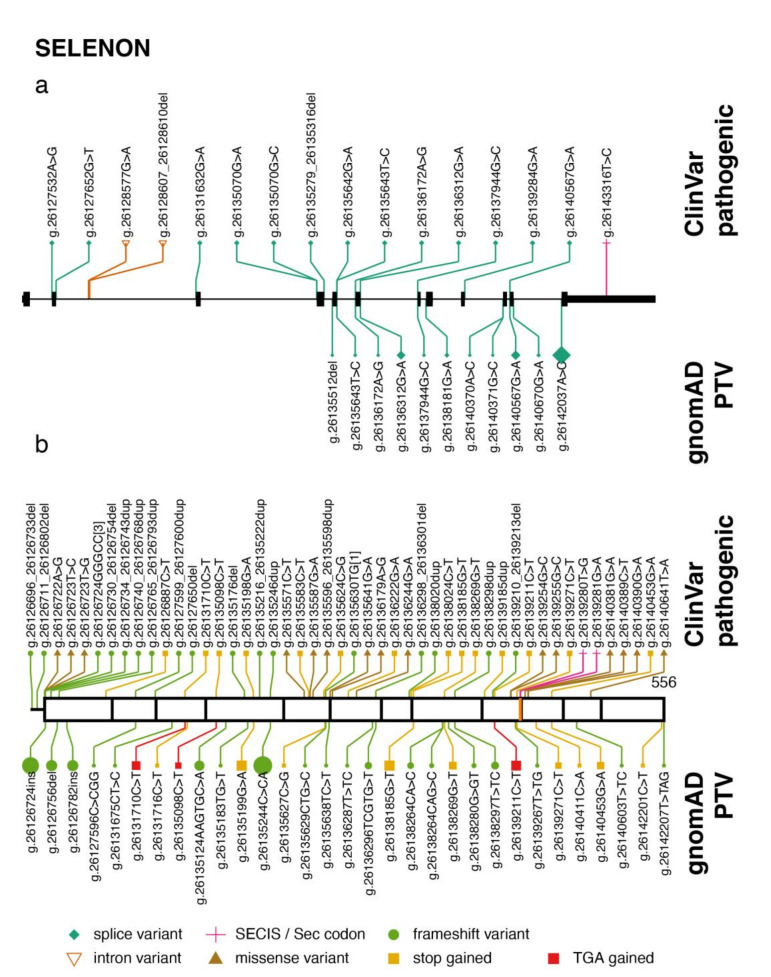
Pathogenic variants and protein-truncating variants in *SELENON.* (**a**) Genomic organization of *SELENON* exons, and location of ClinVar pathogenic variants (above) and gnomAD protein-truncating variants (PTV) (below). The shape and color of each variant corresponds to its predicted consequence (see legend). For gnomAD variants only, the size of the symbol is proportional to the allele frequency. The genomic notation is used to describe each variant. (**b**) Location of variants along the SELENON protein sequence. The length of the protein is indicated on the right. Vertical black lines correspond to exon boundaries, and the vertical orange line correspond to the Sec residue. The same aesthetics for variants as (**a**) are used. The genomic coordinates correspond to genome build GRCh37/hg19; the *SELENON* gene structure and protein sequence correspond to transcript ENST00000374315.

The gnomAD database contains 30 of the pathogenic or likely pathogenic variants, which include both missense and protein-truncating variants (PTV: frameshift, stop gain, and splice variants) (Figure 1). Though they can be considered rare, those included in gnomAD are, presumably, the most common SELENON-RM associated variants in the general population. Their allele frequencies range from 0.000004 to 0.0002, and carriers are all heterozygotes. Some of the variants are more frequent in certain populations. For example, the frameshift variant p.Asn204LysfsTer63 (g. 26135244C>CA) had an allele frequency of 0.001353 in the Ashkenazi Jewish population. In addition to those listed in ClinVar, other PTVs are present in gnomAD (Figure 1), which have not been assessed for clinical significance. Given that all but one of the PTVs that have been submitted to ClinVar are pathogenic (19 in total), it raises the question whether the additional 23 truncating variants in gnomAD could potentially cause SELENON-RM.

### 2.2. GPX4

GPX4 is unique among the five glutathione peroxidases that depend on selenium in humans due to its ability to reduce lipid hydroperoxides and to use protein thiols as donors of electrons in addition to glutathione [33,34]. GPX4 also functions as a major regulator of ferroptosis, a form of regulated cell death characterized by the accumulation of lipid hydroperoxides [35,36]. Gpx4 is essential for embryonic mouse development [6,7], and its deficiency in mice leads to neuronal degeneration, ataxia, and seizures [37].

GPX4 is associated with Sedaghatian-type spondylometaphyseal dysplasia (SSDM). The syndrome was described in 1980 as a congenital autosomal recessive disorder [38], and more recently, inactivating variants in GPX4 were identified in SSDM patients [39]. Patients show skeletal disorder and brain atrophy and die shortly after birth due to respiratory failure [40].

**Figure 2:**
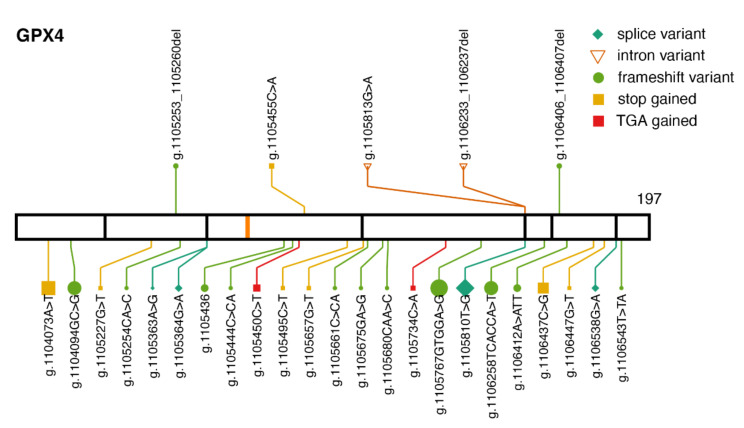
Pathogenic variants and protein-truncating variants in *GPX4.* Location of ClinVar pathogenic variants (above) and gnomAD protein-truncating variants (below), along the GPX4 protein sequence. The same aesthetics as in Figure 1b are used. GPX4 transcript: ENST00000354171.

There are five pathogenic or likely pathogenic variants in GPX4 associated with SSDM in ClinVar (Figure 2). Three of them were reported in [39], while for the other two (p.Gly51_His52insTer and p.Ile170fs) there is no study citation in ClinVar. Only one of the pathogenic variants is observed in gnomAD, c.476 + 5G>A (g. 1105813G>A), with allele frequency 0.00003586. Additional protein-truncating variants in GPX4 are observed in gnomAD, which are not present in ClinVar. Their allele frequencies range from 0.000004 to 0.000065.

### 2.3. Thioredoxin Reductases

Thioredoxin reductases (TXNRD) are oxidoreductases that play a major role in the disulfide reduction system of the cell by converting thioredoxins to their reduced state. There are three TXNRDs in mammals, with different cellular or tissue localization. TXNRD1 is localized mainly in the cytosol and nucleus throughout different tissues, TXNRD2 is localized in the mitochondria also throughout tissues, and TXNRD3 is highly expressed in the testis [2]. All three proteins have the carboxy-terminal motif GCUG that contains the Sec residue [1]. Both Txnrd1 and Txnrd2 are essential for embryonic development in mice [8,9]. TXNRD2 has been associated with two different, unrelated diseases: dilated cardiomyopathy [41] and familial glucocorticoid deficiency [42]. TXNRD1 has been associated with generalized epilepsy [43].

Two variants in TXNRD2, p.Ala59Thr and p.Gly375Arg, were identified in patients that suffered from dilated cardiomyopathy [41]. The clinical significance of p.Gly375Arg is currently uncertain in ClinVar, although it is predicted to be deleterious by the algorithms Polyphen and SIFT, used in gnomAD. The missense variant p.Gly375Arg (c.1123G>A, allele frequency (AF) = 0.000016; and c.1123G>C, AF = 0.00003) is observed in five heterozygous subjects in gnomAD. Only one of them is part of the control set, therefore it is not possible to assume that they are all healthy. A change to glutamic acid in this position is also present in gnomAD, p.Gly375Glu (AF = 0.000004). The other variant associated with dilated cardiomyopathy, p.Ala59Thr, is not present in ClinVar, but is also observed in gnomAD (AF = 0.000004). In this position, a change to proline is also observed, p.Ala59Pro, with an allele frequency of 0.00001. All missense variants in position 59 are heterozygous.

**Figure 3:**
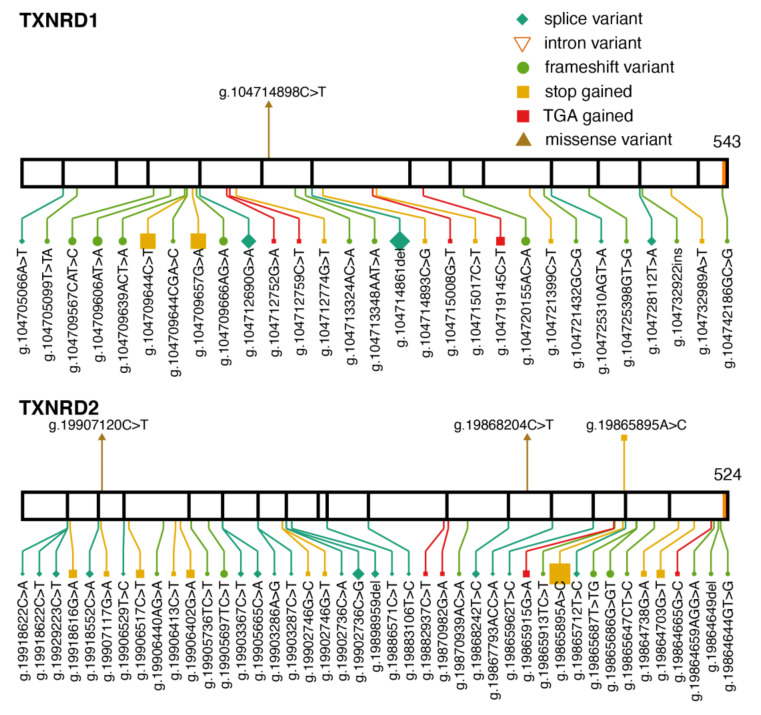
Pathogenic variants and protein-truncating variants in TXNRD1 and TXNRD2. Location of pathogenic variants (above) and gnomAD protein-truncating variants (below) along the TXNRD1 and TXNRD2 protein sequence. The same aesthetics as Figure 1b are used. TXNRD1 transcript: ENST00000526390; TXNRD2 transcript: ENST00000400521.

The variant p.Y447Ter (g.19865895A>C) in TXNRD2 introduces a premature UAG stop codon leading to a truncated protein that lacks the Sec residue. The homozygous form of this variant was identified in several members of a consanguineous Kashmiri family affected with familial glucocorticoid deficiency [42]. The absence of cardiomyopathy in the family was surprising because it implied clinical heterogeneity associated with TXNRD2. The variant was also observed in a heterozygous patient with dilated cardiomyopathy [44]. The global allele frequency in gnomAD is 0.0005 (Figure 3), observed in 141 heterozygotes, but the variant appears to be much more common in the South Asian population, with an allele frequency of 0.0035.

TXNRD1 has been associated with genetic generalized epilepsy. The homozygous variant p.Pro190Leu (g.104714898C>T) was shown to cause decreased TXNRD1 protein levels and turnover rate, and segregated with the disease in a family [43]. The variant is found in one heterozygous subject in gnomAD (AF = 0.000004).

### 2.4. SELENOI

**Figure 4:**
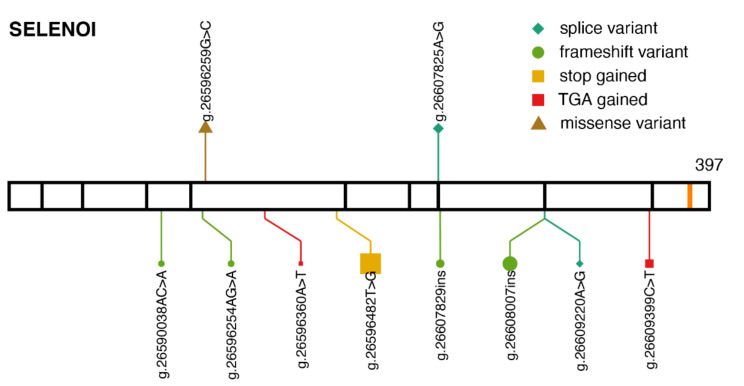
Pathogenic variants and protein-truncating variants in *SELENOI.* Location of ClinVar pathogenic variants (above) and gnomAD protein-truncating variants (below), along the SELENOI protein sequence. The same aesthetics as Figure 1b are used. *SELENOI* transcript: ENST00000260585.

Selenoprotein I (SELENOI; also known as EPT1, Ethanolamine phosphotransferase 1) has been recently added to the list of essential selenoproteins in mice [11]. In humans, two pathogenic variants are currently known. They cause spastic paraplegia, described in two different families [45,46]. The missense variant p.Arg112Pro (g.26596259G>C) [45] hits a highly conserved arginine residue within the CDP-alcohol phosphatidyltransferase (Pfam PF01066). The splice variant g.26607825A>G leads to aberrantly spliced transcripts with exons 6 and 8 affected [46]. The mode of inheritance is autosomal recessive, all patients were homozygous, and unaffected direct relatives were heterozygous. Those two variants are not present in gnomAD (Figure 4), and presumably, are very rare in the population. In concordance with the importance of SELENOI, a strong selection against protein-truncating variants was observed in humans [47].

## 3. Pathogenic Variants Disrupting Selenoprotein Synthesis

### 3.1. TRU-TCA1-1

The Sec-specific tRNA (tRNA^[Ser]Sec^), encoded by *TRU-TCA1-1*, plays a central role in the synthesis of selenoproteins. The Sec tRNA provides the backbone for the biosynthesis of Sec [48], and its anticodon recognizes context-dependent UGA codons to specify Sec insertion [49]. Its deletion in mice (encoded by *Trsp*) is embryonic lethal [50]. Several mouse models have been developed to study its role in health [51]. The Sec tRNA has unique features that distinguish it from other tRNAs: it is the longest tRNA with 90 nucleotides compared to ~75 in other tRNAs; it has a unique structure, with a long acceptor 9-base pairs (bp) stem, a long 6-bp D stem, and an unusually long variable arm [52]. It is transcribed by RNA Pol III, like other tRNAs, but has unique promoter elements [53]. The mature tRNA contains a few modified bases [49], and the tRNA pool is composed of two major isoforms, containing either 5-methoxycarbonylmethyluridine (mcm^5^U) or 5-methoxycarbonylmethyl-2′-O-methyluridine (mcm^5^Um) at position 34 [54]. The presence of mcm^5^Um governs the expression of stress-related selenoproteins [55].

The single nucleotide change C65G was identified in patients [56,57]. The first patient described [56] exhibited a similar clinical phenotype to that observed in SECISBP2 mutation patients. Primary cells from the proband showed low Sec tRNA^Ser[Sec]^ expression with a reduction in mcm^5^Um levels and decreased i6A modification in position 37. This suggests that the tRNA post-transcriptional maturation was impaired. The selenoprotein expression profile showed a deficiency of stress-related selenoprotein levels, but preserved the levels of housekeeping selenoproteins. The position C65 is located in the acceptor arm, adjacent to C64 in the TΨC arm, which interacts with SEPSECS [58]. The precise mechanism leading to the imbalance of Sec tRNA^Ser[Sec]^ isoforms is unclear, with impaired post-transcriptional maturation or unstable interaction with SEPSECS being possibilities [16]. The variant C65G was not observed in gnomAD, but in that same position, the variant C65T was observed in two subjects, both heterozygotes. In total, 83 variants in 55 sites of *TRU-TCA1-1* are currently present in gnomAD, with allele frequencies ranging from 0.000007 to 0.0008. All variants are heterozygous, except for one: the change C28T, in the anticodon arm is observed in two homozygous subjects. Given that many heterozygous variants exist in the general population, it would be reasonable to assume that a single wild type *TRU-TCA1-1* allele is enough to maintain adequate expression of selenoproteins, as observed in heterozygotes for the variant C65G [56]. Remarkably, two subjects have a variant in position 35 of the anticodon triplet. One variant is C35T, which produces a TTA anticodon that is complementary to the stop codon UAA. The other one is C35G, producing a TGA anticodon, which is a Ser anticodon. Both subjects are heterozygous, but the change in the anticodon could have consequences not only on the synthesis of selenoproteins, but potentially on the entire proteome.

### 3.2. SECISBP2

The SECIS binding protein 2 (SECISBP2) binds the SECIS element in the 3′UTR of selenoprotein mRNAs, and interacts with EEFSEC, tRNA^[Ser]Sec^, and the ribosome, to incorporate Sec into the growing peptide. It is an obligate limiting factor for selenoprotein synthesis [59], and it is an essential gene in mice [60]. Its deficiency disrupts the synthesis of selenoproteins, which, in humans, is manifested by a multisystem disorder characterized by low circulating selenium and abnormal thyroid hormone levels [16]. A total of 18 pathogenic variants have been identified in 13 individuals from 11 families [16]. Three of the subjects are homozygous, and the rest are compound heterozygous. Most variants produce a truncated protein, either by stop gain or frameshift, and three are missense variants.

The first 400 N-terminal amino acids of the human SECISBP2 protein are dispensable for Sec incorporation [59,61]. The *C*-terminal region (positions 399 to 784) comprises two domains, the Sec incorporation domain (SID), and the RNA binding domain (RBD) [62]. The RBD domain contains an L7Ae RNA-binding domain that interacts with the SECIS and the 28S ribosomal RNA [63]. Mouse models carrying human mutations have been developed to study the effect of specific pathogenic variants that affect either the RBD or SID regions [64]. The results showed that the variant in the RBD domain abrogates Sec insertion, while in the SID domain, the particular variant tested results in residual SECISBP2 activity in the brain, but it rendered the protein unstable in a tissue-specific manner, being completely degraded in mouse liver.

**Figure 5:**
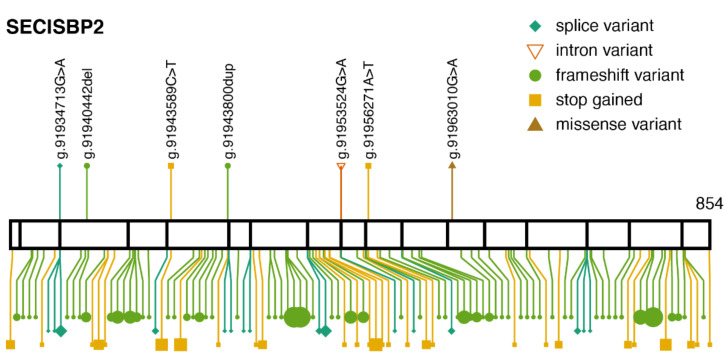
Pathogenic variants and protein-truncating variants in *SECISBP2.* Location of ClinVar pathogenic variants (above) and gnomAD protein-truncating variants (below), along the SECISBP2 protein sequence. The same aesthetics as Figure 1b are used. *SECISBP2* transcript: ENST00000375807.

ClinVar lists seven variants classified as pathogenic/likely pathogenic (Figure 5). Four of them are observed in gnomAD, all with an allele frequency below 0.00003. In addition, 93 protein-truncating variants are observed in gnomAD, which are not listed in ClinVar. They are all heterozygous and their allele frequencies are below 0.00009.

A paralog of SECISBP2, named SECISBP2L, is found in vertebrates [65]. Its function has not been elucidated, but it was shown to lack Sec incorporation activity [66]. Based on gnomAD, SECISBP2L has strong selective constraints against truncating variants [47]. Mice lacking Secisbp2l have been phenotyped and deposited on the International Mouse Phenotyping Consortium (IMPC). Deletion of Secisbp2l has, apparently, no effect on viability, but it shows significant phenotypes in body size, metabolism/adipose tissue, cardiovascular system, skeleton, hearing, and vision (https://www.mousephenotype.org/data/genes/MGI:1917604, accessed on 17 October 2021). However, deletion of Secisbp2 is also reported to have no effect on viability (https://www.mousephenotype.org/data/genes/MGI:1922670, accessed on 17 October 2021), which is in contradiction with the previous body of work that reported Secisbp2 as essential [60].

### 3.3. SEPSECS

SEPSECS catalyzed the conversion of Ser-tRNA^[Ser]Sec^ to Sec-tRNA^[Ser]Sec^, the final step in the biosynthesis of selenocysteine [67]. The crystal structure of human SEPSECS in complex with tRNA^[Ser]Sec^ has been solved [58], providing substantial information on its function. The interaction of SEPSECS and tRNA^[Ser]Sec^ occurs through the tRNA long acceptor-TΨC arms. There is no published study on mice *Sepsecs* knockout, but its deletion is reported as embryonic lethal, with significant phenotypes in heterozygotes including cardiovascular, pigmentation, and vision (https://www.mousephenotype.org/data/genes/MGI:1098791, accessed on 17 October 2021).

Deficiency of SEPSECS in humans causes pontocerebellar hypoplasia 2D (PCH2D), a neurological condition characterized by neurodegeneration and epilepsy. Multiple families from diverse ethnic backgrounds carry homozygous or compound heterozygous mutations that show similar clinical characteristics [16,68].

**Figure 6:**
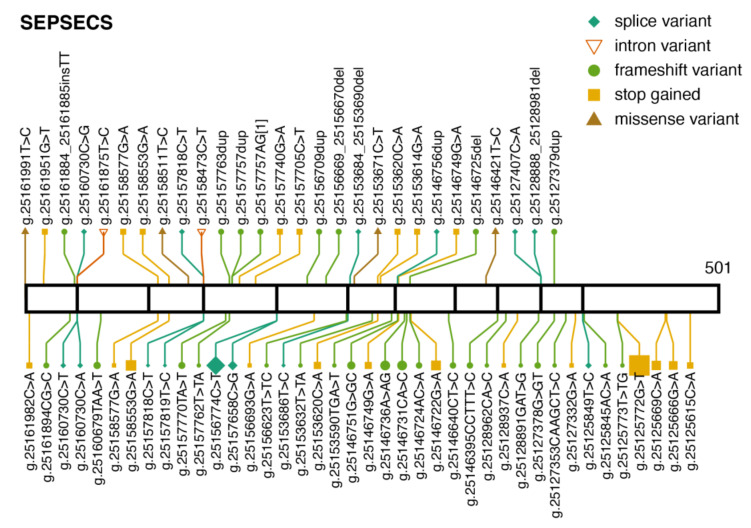
Pathogenic variants and protein-truncating variants in *SEPSECS.* Location of ClinVar pathogenic variants (above) and gnomAD protein-truncating variants (below) along the SEPSECS protein sequence. The same aesthetics as Figure 1b are used. *SEPSECS* transcript: ENST00000382103.

ClinVar currently lists 27 pathogenic or likely pathogenic variants in SEPSECS (Figure 6). Most are protein-truncating variants, and three of them are missense variants. Eleven of those are present in gnomAD and their global allele frequencies are below 0.00004. There are many observed protein-truncating variants observed in gnomAD, which are not present in ClinVar, and have no clinical significance category. The most common is p.Tyr429Ter (g.25125772G>T), which has been observed in three unrelated patients in Finland [69]. All three were compound heterozygous for the same two variants, p.Tyr429Ter and p.Thr325Ser. p.Tyr429Ter is reportedly enriched in the population in Finland [69]. In gnomAD, both variants are observed exclusively within the Finnish population, where the allele frequencies are 0.002791 for p.Tyr429Ter, and 0.0002792 for p.Thr325Ser. The second most common truncating SEPSECS variant in gnomAD is g.25155152C>T, which disrupts the canonical splice acceptor site in intron 4. Interestingly, this variant appears to be also enriched in the Finnish population, with allele frequency 0.002192. Among the 58 subjects in gnomAD, only three are non-Finnish. ClinVar lists its clinical significance as uncertain as it has not been reported as pathogenic or benign. These observations raise the question whether the Finnish population is enriched with pathogenic variants in SEPSECS.

## 4. Common Genetic Variance

Genetic variation is often shared among many individuals in a population. This common variation reflects coinheritance of haplotypes. Functional annotation of haplotypes (groups of single nucleotide variants) is needed to understand hereditary factors linked to complex disease. Genome-wide association studies (GWAS) are designed to find associations between common genetic variants and particular traits.

The NHGRI-EBI GWAS catalog (https://www.ebi.ac.uk/gwas, accessed on 17 October 2021) is a repository of genetic variant-trait associations from published studies [70]. The catalog currently lists 24 associations involving variants in selenoprotein genes. Most of those variants fall in introns and it is not clear what impact in protein function they might produce, or even whether they are the causal variants. Three of them are missense and produce an amino acid change in three selenoproteins. The variant rs225014 (p.Thr92Ala) in DIO2 has been shown to be involved in insulin resistance [71], thyroid functionality [72,73] and the onset and progression of osteoarthritis [74] in humans. DIO2 activates the prohormone thyroxine (T4) into the active thyroid hormone 3,3′,5-triiodothyronine (T3) [2]. The variant p.Thr92Ala is particularly common, with an allele frequency of 0.41. The variant rs5771225 (p.Val3Ala) in SELENOO is associated with late onset Alzheimer’s disease [75] and has an allele frequency of 0.22. SELENOO is a pseudokinase that transfers AMP from ATP to Ser, Thr, and Tyr residues (AMPylation) [76]. Lastly, the variant rs1050450 (p.Pro200Leu) in GPX1, with an allele frequency of 0.28, is associated with decreased hemoglobin levels. Its clinical significance is currently classified as benign according to ClinVar.

Four GWAS on circulating selenium concentrations have been published in recent years [77,78,79,80]. Perhaps not surprisingly, individual variants in selenoproteins did not reach genome-wide significance levels. The authors discuss that though the selenium measurements used (circulating and toenail selenium) are an accepted selenium level biomarker, the selenium concentration might not reflect its functional significance in selenium-sufficient populations [79]. Nonetheless, a locus overlapping genes involved in metabolism of sulfur-containing amino acids reached significance level in two independent cohorts [77,79]. The region overlaps with genes dimethylglycine dehydrogenase (DMGDH) and betaine-homocysteine S-methyltransferase (BHMT), both involved in conversion of homocysteine to methionine. A prospective GWAS also found strong association with greater increase of circulating selenium after supplementation on the same locus in chromosome 5 [80]. These studies revealed a link between selenium exposure and homocysteine metabolism.

The main source of selenium and other essential trace elements in humans is through the diet. Studies on bioavailability in plants and animals suggest that selenium levels vary widely across world regions, with several countries containing relatively low selenium, notably in some parts of China [81,82]. To explore how human populations have adapted to varying levels of dietary selenium levels during history, the signatures of positive selection were assessed by Castellano and collaborators through a survey using genetic polymorphisms in selenoproteins, Cys-containing homologs, and Sec synthesis machinery factors, in 50 human populations [83]. The strongest signals were observed in populations from China. The genes with largest contributions included selenoproteins DIO2, SELENOS, GPX1, SELENOM and SELENOF, and the Sec machinery factors SEPHS2 and SEPSECS. Several single nucleotide polymorphism with known functional consequences showed high levels of population differentiation, including the missense substitution p.Thr92Ala in DIO2 [84], and in GPX1, the missense variant p.Pro200Leu, and a noncoding change A to G (rs3811699) in its promoter region. Positive selection in GPX1 in human populations has been reported also in other studies [85,86].

## 5. Conclusions

Selenocysteine is a genetic trait shared by bacteria, archaea, and eukaryotes. Its origin maps at the root of the tree of life. Throughout evolution, some organisms lost the ability to synthesize selenoproteins and replaced selenocysteine in essential proteins with cysteine, which contains sulfur instead of selenium. Non-selenoprotein cysteine-containing homologs exist for almost all known selenoproteins; they are less costly for the cell to synthesize, but are preferred when it comes to catalysis. It is not fully understood what the advantage of Sec over Cys is. Nevertheless, the presence of 25 selenoproteins in the human genome should convey the requirement for the unique properties of Sec that cannot be compensated by the use of Cys.

Elucidation of the molecular biology behind the insertion of Sec into proteins in response to UGA, paved the way for the identification of a genetic association between selenoproteins and human disease twenty years ago. Since then, several disorders caused by selenoprotein deficiency have been described. Their clinical phenotypes are diverse and different systems are affected, which is not surprising, given that selenoproteins carry out diverse functions and are expressed in different tissues. Moreover, global deficiency of selenoproteins caused by defects in the synthesis machinery result in more complex phenotypes. More surprising, however, is the fact that there are the differences observed between patients with defects in different components of the Sec machinery, SECISBP2 and *TRU-TCA1-1* affect mainly thyroid function, muscle, and growth, while SEPSECS affects the brain and causes a more severe phenotype.

A subset of the observed stop gain variants introduce a UGA codon. This is a special type of genetic variation when it occurs in selenoprotein genes because selenoprotein mRNAs carrying additional UGA codons may produce proteins with extra Sec residues. Sec insertion can occur in multiple sites [87,88], which may be partially functional or have a negative gain of function. mRNAs with early termination codons can be targeted by nonsense-mediated decay (NMD) to prevent its translation. But we previously showed that, in case of selenoproteins, NMD is less efficient if the stop gained is UGA, compared to the other two stop codons [47], which may increase insertion of extra Sec residues. TGA gain is observed in multiple sites in all selenoproteins included in this review and are indicated in the corresponding figures.

The advent of genome sequencing, particularly exome sequencing for clinical diagnosis, has led to the identification of dozens of variants that cause disease through deficiency of selenoproteins, either by inactivating individual selenoprotein genes or by disrupting the selenoprotein synthesis pathway. Initiatives like ClinVar and gnomAD have become instrumental for researchers and clinicians by giving access to a vast amount of genomic data, and will help accelerate our understanding of the associations between genetic variation and health. Undoubtedly, more pathogenic variants in selenoproteins will be discovered, which will provide insights into the function of selenoproteins and opportunities to better understand the role of selenium in human health and disease.

## References

[1] Mariotti M., Ridge P.G., Zhang Y., Lobanov A.V., Pringle T.H., Guigo R., Hatfield D.L., Gladyshev V.N. (2012). Composition and Evolution of the Vertebrate and Mammalian Selenoproteomes. PLoS ONE.

[2] Labunskyy V.M., Hatfield D.L., Gladyshev V.N. (2014). Selenoproteins: Molecular Pathways and Physiological Roles. Physiol. Rev..

[3] Allmang C., Wurth L., Krol A. (2009). The Selenium to Selenoprotein Pathway in Eukaryotes: More Molecular Partners than Anticipated. Biochim. Biophys. Acta.

[4] Kryukov G.V., Castellano S., Novoselov S.V., Lobanov A.V., Zehtab O., Guigó R., Gladyshev V.N. (2003). Characterization of Mammalian Selenoproteomes. Science.

[5] Conrad M., Schweizer U., Hatfield D.L., Schweizer U., Tsuji P.A., Gladyshev V.N. (2016). Mouse Models That Target Individual Selenoproteins. Selenium.

[6] Yant L.J., Ran Q., Rao L., Van Remmen H., Shibatani T., Belter J.G., Motta L., Richardson A., Prolla T.A. (2003). The Selenoprotein GPX4 Is Essential for Mouse Development and Protects from Radiation and Oxidative Damage Insults. Free Radic. Biol. Med..

[7] Imai H., Hirao F., Sakamoto T., Sekine K., Mizukura Y., Saito M., Kitamoto T., Hayasaka M., Hanaoka K., Nakagawa Y. (2003). Early Embryonic Lethality Caused by Targeted Disruption of the Mouse PHGPx Gene. Biochem. Biophys. Res. Commun..

[8] Jakupoglu C., Przemeck G.K.H., Schneider M., Moreno S.G., Mayr N., Hatzopoulos A.K., de Angelis M.H., Wurst W., Bornkamm G.W., Brielmeier M. (2005). Cytoplasmic Thioredoxin Reductase Is Essential for Embryogenesis but Dispensable for Cardiac Development. Mol. Cell. Biol..

[9] Conrad M., Jakupoglu C., Moreno S.G., Lippl S., Banjac A., Schneider M., Beck H., Hatzopoulos A.K., Just U., Sinowatz F. (2004). Essential Role for Mitochondrial Thioredoxin Reductase in Hematopoiesis, Heart Development, and Heart Function. Mol. Cell. Biol..

[10] Boukhzar L., Hamieh A., Cartier D., Tanguy Y., Alsharif I., Castex M., Arabo A., El Hajji S., Bonnet J.-J., Errami M. (2016). Selenoprotein T Exerts an Essential Oxidoreductase Activity That Protects Dopaminergic Neurons in Mouse Models of Parkinson’s Disease. Antioxid. Redox Signal..

[11] Avery J.C., Yamazaki Y., Hoffmann F.W., Folgelgren B., Hoffmann P.R. (2020). Selenoprotein I Is Essential for Murine Embryogenesis. Arch. Biochem. Biophys..

[12] Landrum M.J., Chitipiralla S., Brown G.R., Chen C., Gu B., Hart J., Hoffman D., Jang W., Kaur K., Liu C. (2020). ClinVar: Improvements to Accessing Data. Nucleic Acids Res..

[13] Karczewski K.J., Francioli L.C., Tiao G., Cummings B.B., Alföldi J., Wang Q., Collins R.L., Laricchia K.M., Ganna A., Birnbaum D.P. (2020). The Mutational Constraint Spectrum Quantified from Variation in 141,456 Humans. Nature.

[14] Schweizer U., Fradejas-Villar N. (2016). Why 21? The Significance of Selenoproteins for Human Health Revealed by Inborn Errors of Metabolism. FASEB J..

[15] Fradejas-Villar N. (2018). Consequences of Mutations and Inborn Errors of Selenoprotein Biosynthesis and Functions. Free Radic. Biol. Med..

[16] Schoenmakers E., Chatterjee K. (2020). Human Disorders Affecting the Selenocysteine Incorporation Pathway Cause Systemic Selenoprotein Deficiency. Antioxid. Redox Signal..

[17] Schweizer U., Bohleber S., Zhao W., Fradejas-Villar N. (2021). The Neurobiology of Selenium: Looking Back and to the Future. Front. Neurosci..

[18] Castets P., Lescure A., Guicheney P., Allamand V. (2012). Selenoprotein N in Skeletal Muscle: From Diseases to Function. J. Mol. Med..

[19] Lescure A., Gautheret D., Carbon P., Krol A. (1999). Novel Selenoproteins Identified in Silico and in Vivo by Using a Conserved RNA Structural Motif. J. Biol. Chem..

[20] Moghadaszadeh B., Petit N., Jaillard C., Brockington M., Quijano Roy S., Merlini L., Romero N., Estournet B., Desguerre I., Chaigne D. (2001). Mutations in SEPN1 Cause Congenital Muscular Dystrophy with Spinal Rigidity and Restrictive Respiratory Syndrome. Nat. Genet..

[21] Jurynec M.J., Xia R., Mackrill J.J., Gunther D., Crawford T., Flanigan K.M., Abramson J.J., Howard M.T., Grunwald D.J. (2008). Selenoprotein N Is Required for Ryanodine Receptor Calcium Release Channel Activity in Human and Zebrafish Muscle. Proc. Natl. Acad. Sci. USA.

[22] Petit N., Lescure A., Rederstorff M., Krol A., Moghadaszadeh B., Wewer U.M., Guicheney P. (2003). Selenoprotein N: An Endoplasmic Reticulum Glycoprotein with an Early Developmental Expression Pattern. Hum. Mol. Genet..

[23] Chernorudskiy A., Varone E., Colombo S.F., Fumagalli S., Cagnotto A., Cattaneo A., Briens M., Baltzinger M., Kuhn L., Bachi A. (2020). Selenoprotein N Is an Endoplasmic Reticulum Calcium Sensor That Links Luminal Calcium Levels to a Redox Activity. Proc. Natl. Acad. Sci. USA.

[24] Rederstorff M., Castets P., Arbogast S., Lainé J., Vassilopoulos S., Beuvin M., Dubourg O., Vignaud A., Ferry A., Krol A. (2011). Increased Muscle Stress-Sensitivity Induced by Selenoprotein N Inactivation in Mouse: A Mammalian Model for SEPN1-Related Myopathy. PLoS ONE.

[25] Castets P., Bertrand A.T., Beuvin M., Ferry A., Le Grand F., Castets M., Chazot G., Rederstorff M., Krol A., Lescure A. (2011). Satellite Cell Loss and Impaired Muscle Regeneration in Selenoprotein N Deficiency. Hum. Mol. Genet..

[26] Moghadaszadeh B., Desguerre I., Topaloglu H., Muntoni F., Pavek S., Sewry C., Mayer M., Fardeau M., Tomé F.M., Guicheney P. (1998). Identification of a New Locus for a Peculiar Form of Congenital Muscular Dystrophy with Early Rigidity of the Spine, on Chromosome 1p35-36. Am. J. Hum. Genet..

[27] Ferreiro A., Quijano-Roy S., Pichereau C., Moghadaszadeh B., Goemans N., Bönnemann C., Jungbluth H., Straub V., Villanova M., Leroy J.-P. (2002). Mutations of the Selenoprotein N Gene, Which Is Implicated in Rigid Spine Muscular Dystrophy, Cause the Classical Phenotype of Multiminicore Disease: Reassessing the Nosology of Early-Onset Myopathies. Am. J. Hum. Genet..

[28] Clarke N.F., Kidson W., Quijano-Roy S., Estournet B., Ferreiro A., Guicheney P., Manson J.I., Kornberg A.J., Shield L.K., North K.N. (2006). SEPN1: Associated with Congenital Fiber-Type Disproportion and Insulin Resistance. Ann. Neurol..

[29] Ferreiro A., Ceuterick-de Groote C., Marks J.J., Goemans N., Schreiber G., Hanefeld F., Fardeau M., Martin J.-J., Goebel H.H., Richard P. (2004). Desmin-Related Myopathy with Mallory Body-like Inclusions Is Caused by Mutations of the Selenoprotein, N. Gene. Ann. Neurol..

[30] Villar-Quiles R.N., von der Hagen M., Métay C., Gonzalez V., Donkervoort S., Bertini E., Castiglioni C., Chaigne D., Colomer J., Cuadrado M.L. (2020). The Clinical, Histologic, and Genotypic Spectrum of SEPN1-Related Myopathy: A Case Series. Neurology.

[31] Allamand V., Richard P., Lescure A., Ledeuil C., Desjardin D., Petit N., Gartioux C., Ferreiro A., Krol A., Pellegrini N. (2006). A Single Homozygous Point Mutation in a 3’untranslated Region Motif of Selenoprotein N MRNA Causes SEPN1-Related Myopathy. EMBO Rep..

[32] Maiti B., Arbogast S., Allamand V., Moyle M.W., Anderson C.B., Richard P., Guicheney P., Ferreiro A., Flanigan K.M., Howard M.T. (2009). A Mutation in the SEPN1 Selenocysteine Redefinition Element (SRE) Reduces Selenocysteine Incorporation and Leads to SEPN1-Related Myopathy. Hum. Mutat..

[33] Ursini F., Maiorino M., Brigelius-Flohé R., Aumann K.D., Roveri A., Schomburg D., Flohé L. (1995). Diversity of Glutathione Peroxidases. Methods Enzymol..

[34] Ursini F., Heim S., Kiess M., Maiorino M., Roveri A., Wissing J., Flohé L. (1999). Dual Function of the Selenoprotein PHGPx during Sperm Maturation. Science.

[35] Ingold I., Berndt C., Schmitt S., Doll S., Poschmann G., Buday K., Roveri A., Peng X., Porto Freitas F., Seibt T. (2018). Selenium Utilization by GPX4 Is Required to Prevent Hydroperoxide-Induced Ferroptosis. Cell.

[36] Stockwell B.R., Friedmann Angeli J.P., Bayir H., Bush A.I., Conrad M., Dixon S.J., Fulda S., Gascón S., Hatzios S.K., Kagan V.E. (2017). Ferroptosis: A Regulated Cell Death Nexus Linking Metabolism, Redox Biology, and Disease. Cell.

[37] Hambright W.S., Fonseca R.S., Chen L., Na R., Ran Q. (2017). Ablation of Ferroptosis Regulator Glutathione Peroxidase 4 in Forebrain Neurons Promotes Cognitive Impairment and Neurodegeneration. Redox Biol..

[38] Sedaghatian M.R. (1980). Congenital Lethal Metaphyseal Chondrodysplasia: A Newly Recognized Complex Autosomal Recessive Disorder. Am. J. Med. Genet..

[39] Smith A.C., Mears A.J., Bunker R., Ahmed A., MacKenzie M., Schwartzentruber J.A., Beaulieu C.L., Ferretti E., FORGE Canada Consortium, Majewski J. (2014). Mutations in the Enzyme Glutathione Peroxidase 4 Cause Sedaghatian-Type Spondylometaphyseal Dysplasia. J. Med. Genet..

[40] Aygun C., Celik F.C., Nural M.S., Azak E., Kucukoduk S., Ogur G., Incesu L. (2012). Simplified Gyral Pattern with Cerebellar Hypoplasia in Sedaghatian Type Spondylometaphyseal Dysplasia: A Clinical Report and Review of the Literature. Am. J. Med. Genet. A.

[41] Sibbing D., Pfeufer A., Perisic T., Mannes A.M., Fritz-Wolf K., Unwin S., Sinner M.F., Gieger C., Gloeckner C.J., Wichmann H.-E. (2011). Mutations in the Mitochondrial Thioredoxin Reductase Gene TXNRD2 Cause Dilated Cardiomyopathy. Eur. Heart J..

[42] Prasad R., Chan L.F., Hughes C.R., Kaski J.P., Kowalczyk J.C., Savage M.O., Peters C.J., Nathwani N., Clark A.J.L., Storr H.L. (2014). Thioredoxin Reductase 2 (TXNRD2) Mutation Associated with Familial Glucocorticoid Deficiency (FGD). J. Clin. Endocrinol. Metab..

[43] Kudin A.P., Baron G., Zsurka G., Hampel K.G., Elger C.E., Grote A., Weber Y., Lerche H., Thiele H., Nürnberg P. (2017). Homozygous Mutation in TXNRD1 Is Associated with Genetic Generalized Epilepsy. Free Radic. Biol. Med..

[44] Rojnueangnit K., Sirichongkolthong B., Wongwandee R., Khetkham T., Noojarern S., Khongkraparn A., Wattanasirichaigoon D. (2020). Identification of Gene Mutations in Primary Pediatric Cardiomyopathy by Whole Exome Sequencing. Pediatr. Cardiol..

[45] Ahmed M.Y., Al-Khayat A., Al-Murshedi F., Al-Futaisi A., Chioza B.A., Fernandez-Murray J.P., Self J.E., Salter C.G., Harlalka G.V., Rawlins L.E. (2017). A Mutation of EPT1 (SELENOI) Underlies a New Disorder of Kennedy Pathway Phospholipid Biosynthesis. Brain.

[46] Horibata Y., Elpeleg O., Eran A., Hirabayashi Y., Savitzki D., Tal G., Mandel H., Sugimoto H. (2018). EPT1 (Selenoprotein I) Is Critical for the Neural Development and Maintenance of Plasmalogen in Humans. J. Lipid Res..

[47] Santesmasses D., Mariotti M., Gladyshev V.N. (2020). Tolerance to Selenoprotein Loss Differs between Human and Mouse. Mol. Biol. Evol..

[48] Turanov A.A., Xu X.-M., Carlson B.A., Yoo M.-H., Gladyshev V.N., Hatfield D.L. (2011). Biosynthesis of Selenocysteine, the 21st Amino Acid in the Genetic Code, and a Novel Pathway for Cysteine Biosynthesis. Adv. Nutr..

[49] Diamond A., Dudock B., Hatfield D. (1981). Structure and Properties of a Bovine Liver UGA Suppressor Serine TRNA with a Tryptophan Anticodon. Cell.

[50] Bösl M.R., Takaku K., Oshima M., Nishimura S., Taketo M.M. (1997). Early Embryonic Lethality Caused by Targeted Disruption of the Mouse Selenocysteine TRNA Gene (Trsp). Proc. Natl. Acad. Sci. USA.

[51] Carlson B.A. (2016). Selenocysteine TRNA[Ser]Sec Mouse Models for Elucidating Roles of Selenoproteins in Health and Development. Selenium.

[52] Itoh Y., Chiba S., Sekine S.-I., Yokoyama S. (2009). Crystal Structure of Human Selenocysteine TRNA. Nucleic Acids Res..

[53] Carlson B.A., Lee B.J., Tsuji P.A., Tobe R., Park J.M., Schweizer U., Gladyshev V.N., Hatfield D.L. (2016). Selenocysteine TRNA[Ser]Sec: From Nonsense Suppressor TRNA to the Quintessential Constituent in Selenoprotein Biosynthesis. Selenium.

[54] Diamond A.M., Choi I.S., Crain P.F., Hashizume T., Pomerantz S.C., Cruz R., Steer C.J., Hill K.E., Burk R.F., McCloskey J.A. (1993). Dietary Selenium Affects Methylation of the Wobble Nucleoside in the Anticodon of Selenocysteine TRNA([Ser]Sec). J. Biol. Chem..

[55] Carlson B.A., Moustafa M.E., Sengupta A., Schweizer U., Shrimali R., Rao M., Zhong N., Wang S., Feigenbaum L., Lee B.J. (2007). Selective Restoration of the Selenoprotein Population in a Mouse Hepatocyte Selenoproteinless Background with Different Mutant Selenocysteine TRNAs Lacking Um34. J. Biol. Chem..

[56] Schoenmakers E., Carlson B., Agostini M., Moran C., Rajanayagam O., Bochukova E., Tobe R., Peat R., Gevers E., Muntoni F. (2016). Mutation in Human Selenocysteine Transfer RNA Selectively Disrupts Selenoprotein Synthesis. J. Clin. Investig..

[57] Geslot A., Savagner F., Caron P. (2021). Inherited Selenocysteine Transfer RNA Mutation: Clinical and Hormonal Evaluation of 2 Patients. Eur. Thyroid J..

[58] Palioura S., Sherrer R.L., Steitz T.A., Söll D., Simonovic M. (2009). The Human SepSecS-TRNASec Complex Reveals the Mechanism of Selenocysteine Formation. Science.

[59] Copeland P.R., Fletcher J.E., Carlson B.A., Hatfield D.L., Driscoll D.M. (2000). A Novel RNA Binding Protein, SBP2, Is Required for the Translation of Mammalian Selenoprotein MRNAs. EMBO J..

[60] Seeher S., Atassi T., Mahdi Y., Carlson B.A., Braun D., Wirth E.K., Klein M.O., Reix N., Miniard A.C., Schomburg L. (2014). Secisbp2 Is Essential for Embryonic Development and Enhances Selenoprotein Expression. Antioxid. Redox Signal..

[61] Mehta A., Rebsch C.M., Kinzy S.A., Fletcher J.E., Copeland P.R. (2004). Efficiency of Mammalian Selenocysteine Incorporation. J. Biol. Chem..

[62] Copeland P.R., Stepanik V.A., Driscoll D.M. (2001). Insight into Mammalian Selenocysteine Insertion: Domain Structure and Ribosome Binding Properties of Sec Insertion Sequence Binding Protein 2. Mol. Cell. Biol..

[63] Allmang C., Carbon P., Krol A. (2002). The SBP2 and 15.5 KD/Snu13p Proteins Share the Same RNA Binding Domain: Identification of SBP2 Amino Acids Important to SECIS RNA Binding. RNA.

[64] Zhao W., Bohleber S., Schmidt H., Seeher S., Howard M.T., Braun D., Arndt S., Reuter U., Wende H., Birchmeier C. (2019). Ribosome Profiling of Selenoproteins in Vivo Reveals Consequences of Pathogenic Secisbp2 Missense Mutations. J. Biol. Chem..

[65] Donovan J., Copeland P.R. (2012). Selenocysteine Insertion Sequence Binding Protein 2L Is Implicated as a Novel Post-Transcriptional Regulator of Selenoprotein Expression. PLoS ONE.

[66] Donovan J., Copeland P.R. (2009). Evolutionary History of Selenocysteine Incorporation from the Perspective of SECIS Binding Proteins. BMC Evol. Biol..

[67] Small-Howard A., Morozova N., Stoytcheva Z., Forry E.P., Mansell J.B., Harney J.W., Carlson B.A., Xu X.-M., Hatfield D.L., Berry M.J. (2006). Supramolecular Complexes Mediate Selenocysteine Incorporation in Vivo. Mol. Cell. Biol..

[68] Schoenmakers E., Schoenmakers N., Chatterjee K., Hatfield D.L., Berry M.J., Gladyshev N.V. (2016). Mutations in Humans That Adversely Affect the Selenoprotein Synthesis Pathway. Selenium. Its Molecular Biology and Role in Human Health.

[69] Anttonen A.-K., Hilander T., Linnankivi T., Isohanni P., French R.L., Liu Y., Simonović M., Söll D., Somer M., Muth-Pawlak D. (2015). Selenoprotein Biosynthesis Defect Causes Progressive Encephalopathy with Elevated Lactate. Neurology.

[70] Buniello A., MacArthur J.A.L., Cerezo M., Harris L.W., Hayhurst J., Malangone C., McMahon A., Morales J., Mountjoy E., Sollis E. (2019). The NHGRI-EBI GWAS Catalog of Published Genome-Wide Association Studies, Targeted Arrays and Summary Statistics 2019. Nucleic Acids Res..

[71] Mentuccia D., Proietti-Pannunzi L., Tanner K., Bacci V., Pollin T.I., Poehlman E.T., Shuldiner A.R., Celi F.S. (2002). Association between a Novel Variant of the Human Type 2 Deiodinase Gene Thr92Ala and Insulin Resistance: Evidence of Interaction with the Trp64Arg Variant of the Beta-3-Adrenergic Receptor. Diabetes.

[72] Castagna M.G., Dentice M., Cantara S., Ambrosio R., Maino F., Porcelli T., Marzocchi C., Garbi C., Pacini F., Salvatore D. (2017). DIO2 Thr92Ala Reduces Deiodinase-2 Activity and Serum-T3 Levels in Thyroid-Deficient Patients. J. Clin. Endocrinol. Metab..

[73] Park E., Jung J., Araki O., Tsunekawa K., Park S.Y., Kim J., Murakami M., Jeong S.-Y., Lee S. (2018). Concurrent TSHR Mutations and DIO2 T92A Polymorphism Result in Abnormal Thyroid Hormone Metabolism. Sci. Rep..

[74] Meulenbelt I., Min J.L., Bos S., Riyazi N., Houwing-Duistermaat J.J., van der Wijk H.-J., Kroon H.M., Nakajima M., Ikegawa S., Uitterlinden A.G. (2008). Identification of DIO2 as a New Susceptibility Locus for Symptomatic Osteoarthritis. Hum. Mol. Genet..

[75] Mez J., Chung J., Jun G., Kriegel J., Bourlas A.P., Sherva R., Logue M.W., Barnes L.L., Bennett D.A., Buxbaum J.D. (2017). Two Novel Loci, COBL and SLC10A2, for Alzheimer’s Disease in African Americans. Alzheimers. Dement..

[76] Sreelatha A., Yee S.S., Lopez V.A., Park B.C., Kinch L.N., Pilch S., Servage K.A., Zhang J., Jiou J., Karasiewicz-Urbańska M. (2018). Protein AMPylation by an Evolutionarily Conserved Pseudokinase. Cell.

[77] Evans D.M., Zhu G., Dy V., Heath A.C., Madden P.A.F., Kemp J.P., McMahon G., St Pourcain B., Timpson N.J., Golding J. (2013). Genome-Wide Association Study Identifies Loci Affecting Blood Copper, Selenium and Zinc. Hum. Mol. Genet..

[78] Gong J., Hsu L., Harrison T., King I.B., Stürup S., Song X., Duggan D., Liu Y., Hutter C., Chanock S.J. (2013). Genome-Wide Association Study of Serum Selenium Concentrations. Nutrients.

[79] Cornelis M.C., Fornage M., Foy M., Xun P., Gladyshev V.N., Morris S., Chasman D.I., Hu F.B., Rimm E.B., Kraft P. (2015). Genome-Wide Association Study of Selenium Concentrations. Hum. Mol. Genet..

[80] Batai K., Trejo M.J., Chen Y., Kohler L.N., Lance P., Ellis N.A., Cornelis M.C., Chow H.-H.S., Hsu C.-H., Jacobs E.T. (2021). Genome-Wide Association Study of Response to Selenium Supplementation and Circulating Selenium Concentrations in Adults of European Descent. J. Nutr..

[81] Thomson C.D., Burton C.E., Robinson M.F. (1978). On Supplementing the Selenium Intake of New Zealanders. 1. Short Experiments with Large Doses of Selenite or Selenomethionine. Br. J. Nutr..

[82] Xia Y., Hill K.E., Byrne D.W., Xu J., Burk R.F. (2005). Effectiveness of Selenium Supplements in a Low-Selenium Area of China. Am. J. Clin. Nutr..

[83] White L., Romagné F., Müller E., Erlebach E., Weihmann A., Parra G., Andrés A.M., Castellano S. (2015). Genetic Adaptation to Levels of Dietary Selenium in Recent Human History. Mol. Biol. Evol..

[84] Canani L.H., Capp C., Dora J.M., Meyer E.L.S., Wagner M.S., Harney J.W., Larsen P.R., Gross J.L., Bianco A.C., Maia A.L. (2005). The Type 2 Deiodinase A/G (Thr92Ala) Polymorphism Is Associated with Decreased Enzyme Velocity and Increased Insulin Resistance in Patients with Type 2 Diabetes Mellitus. J. Clin. Endocrinol. Metab..

[85] Foster C.B., Aswath K., Chanock S.J., McKay H.F., Peters U. (2006). Polymorphism Analysis of Six Selenoprotein Genes: Support for a Selective Sweep at the Glutathione Peroxidase 1 Locus (3p21) in Asian Populations. BMC Genet..

[86] Engelken J., Espadas G., Mancuso F.M., Bonet N., Scherr A.-L., Jímenez-Álvarez V., Codina-Solà M., Medina-Stacey D., Spataro N., Stoneking M. (2016). Signatures of Evolutionary Adaptation in Quantitative Trait Loci Influencing Trace Element Homeostasis in Liver. Mol. Biol. Evol..

[87] Turanov A.A., Lobanov A.V., Hatfield D.L., Gladyshev V.N. (2013). UGA Codon Position-Dependent Incorporation of Selenocysteine into Mammalian Selenoproteins. Nucleic Acids Res..

[88] Wen W., Weiss S.L., Sunde R.A. (1998). UGA Codon Position Affects the Efficiency of Selenocysteine Incorporation into Glutathione Peroxidase-1. J. Biol. Chem..

